# Supplementary material for: Pyruvate Dehydrogenase Kinase 1 inhibition mediated oxidative phosphorylation enhancement in cartilage promotes osteoarthritis progression
Source: BMC Musculoskelet Disord. 2023 Jul 20;24:597. doi: 10.1186/s12891-023-06585-6 (PMC10357736; doi:10.1186/s12891-023-06585-6)
Supplement: Supplementary file 1 — Additional file 1. [file 12891_2023_6585_MOESM1_ESM.zip › BP.Gsea.1653623667859/heat_map_corr_plot.html]

Heat map and correlation plot for 55457\_collapsed\_to\_symbols.cls.cls#OA\_versus\_normal  

Fig 1: heat\_map      
 Heat Map of the top 50 features for each phenotype in 55457\_collapsed\_to\_symbols.cls.cls#OA\_versus\_normal

  
  

Fig 2: Ranked Gene List Correlation Profile      
 Ranked list correlations for 55457\_collapsed\_to\_symbols.cls.cls#OA\_versus\_normal

  
  
    
